# Supplementary material for: A microtubule‐LUZP1 association around tight junction promotes epithelial cell apical constriction
Source: EMBO J. 2020 Dec 21;40(2):e104712. doi: 10.15252/embj.2020104712 (PMC7809799; doi:10.15252/embj.2020104712)
Supplement: Supplementary file 1 — Appendix [file EMBJ-40-e104712-s001.pdf]

Appendix

Contents

Appendix Table S1 - Cloning primers

Appendix Table S2 - Knockout oligos

Appendix Table S1 – Cloning primers

Primer Name	Sequence
mouse LUZP1_F	caattgATGGCAGAACTCACTAACTAC
mouse LUZP1_R	caattgTCAGTCTTCCTCAGCATAAGG
mouse LUZP1 N 1-353_R	caattgTTACTCTTTTTGTTTCCTGACCT
mouse LUZP1 M 354-706_F	caattgTTAGGAAATGGAGACATAGA
mouse LUZP1 M 354-706_R	caattgTTAAAATAGGGCAGTTCTAGTTC
mouse LUZP1 C 707-1068_F	caattgGAAAATGATAAAAATGCTGC
mouse LUZP1 sequence check f2	ATGTTCCCAGCTTTGTTTGAGCCT
mouse LUZP1 sequence check f3	AAATGCCGTCCTCTATCCGTAG
mouse LUZP1 sequence check f4	ACTCCATGCTCAGGGCCCATCT
mouse LUZP1 sequence check f5	TCTGAAGCCGCTCAATTTCAGATTTTAGC
mouse LUZP1 sequence check f6	AAGCCATAGTGCCTCTTCAGACAGCA
mouse LUZP1 sequence check f7	CTGCTGGTAACAAATGTGCTGTCCT
human MRLC_F	gaattcATGTCGAGCAAAAAGGCAAAGAC
human MRLC_R	gaattcTCAGTCATCTTTGTCTTTGGCTC
mouse PP1cβ/δ_F	ttagtcgacATGGCGGACGGGGAGCTGAA
mouse PP1cβ/δ_R	gatatcTCACCTTTTCTTCGGTGGATTAG

Appendix Table S2 – Knockout oligos

Primer Name	Sequence
mouse LUZP1 KO vector1F	caccGGCAGAACTCACTAACTACA
mouse LUZP1 KO vector1R	aaacTGTAGTTAGTGAGTTCTGCC
mouse LUZP1 KO vector2F	caccGGATGAGCTCCTGGACCTCC
mouse LUZP1 KO vector2R	aaacGGAGGTCCAGGAGCTCATCC
mouse LUZP1 KO vector3F	caccGCTCCTGGACCTCCAGGACA
mouse LUZP1 KO vector3R	aaacTGTCCTGGAGGTCCAGGAGC
mouse ZO-2 KO vector F	caccGGCAGCGCGGTCCAGGCATG
mouse ZO-2 KO vector R	aaacCATGCCTGGACCGCGCTGCC
mouse E-cadherin KO vector F	caccGGTCTACACCTTCCCGGTGC
mouse E-cadherin KO vector R	aaacGCACCGGGAAGGTGTAGACC
