# Supplementary material for: A microtubule‐LUZP1 association around tight junction promotes epithelial cell apical constriction
Source: EMBO J. 2020 Dec 21;40(2):e104712. doi: 10.15252/embj.2020104712 (PMC7809799; doi:10.15252/embj.2020104712)
Supplement: Supplementary file 2 — Expanded View Figures PDF [file EMBJ-40-e104712-s002.pdf]

Expanded View Figures

Figure EV1. LUZP1 is an apical junctional complex (AJC)-enriched and microtubule (MT)-associated protein expressing ubiquitously in various tissues.

A Representative electron micrograph showing the relationships among tight junctions (TJs), adherens junctions (AJs), AJCs, TJ-associated circumferential rings (CRs), AJ-associated CRs, and AJC-associated CRs. Scale bar, 200 nm.

B A schematic drawing of the apical MT network in epithelial cells. Apical MTs are different from classical apicobasal MTs and are associated with TJs in a side-by-side manner.

C Schematics of fractions used in the membrane overlay assay.

D A schematic of LUZP1 regions. We divided LUZP1 into three different regions for subsequent region analyses.

E MT co-sedimentation assays of different GST-LUZP1 regions. N-terminal (red arrowheads) and middle (blue arrowheads) regions of LUZP1 bound MTs whereas the C-terminal (green arrowheads) region did not bound MTs. The non-specific staining was marked by asterisks (*). S, supernatant; P, pellet.

F Characterization of generated antibodies against LUZP1 in cultured epithelial cells. Immunoblots showed that all of the antibodies, generated against N-terminal, middle, and C-terminal, could react with endogenous and exogenous LUZP1, whereas LUZP1 immunoblot signals (red arrowhead) were lost in LUZP1 knockout (LUZP1 KO) epithelial Eph4 cells. Non-specific signals (*, black arrowhead), which did not disappear in LUZP1 KO cells, were also detected in the antibody against LUZP1 N-terminal. MDCK, Madin-Darby canine kidney cells.

G Confirmation of LUZP1 enrichment in an AJC fraction. The fractions of “Pellet after low-speed centrifugation of liver homogenate”, “Bile canaliculus”, and “AJC fraction” were prepared according to previous reports. LUZP1 immunoblot signals (red arrowhead) were hardly detectable in the fraction of “Pellet after low-speed centrifugation of liver homogenate” but were evident in the AJC-enriched bile canaliculus fraction and the AJC fraction. CBB, Coomassie brilliant blue. The non-specific staining was marked by asterisks (*).

H LUZP1 expressions in various tissues. Immunoblots using an antibody against LUZP1-N-terminal showed that LUZP1 ubiquitously expressed across various tissues. The non-specific staining was marked by asterisks (*).

I Co-immunoprecipitation assay of WT Eph4 cells using the anti-LUZP1 antibody. In addition to LUZP1, ZO-1, α-tubulin, and MLC were all included in the immunoprecipitated fraction. IB, immunoblotting.

Source data are available online for this figure.

 © 2021 The Authors

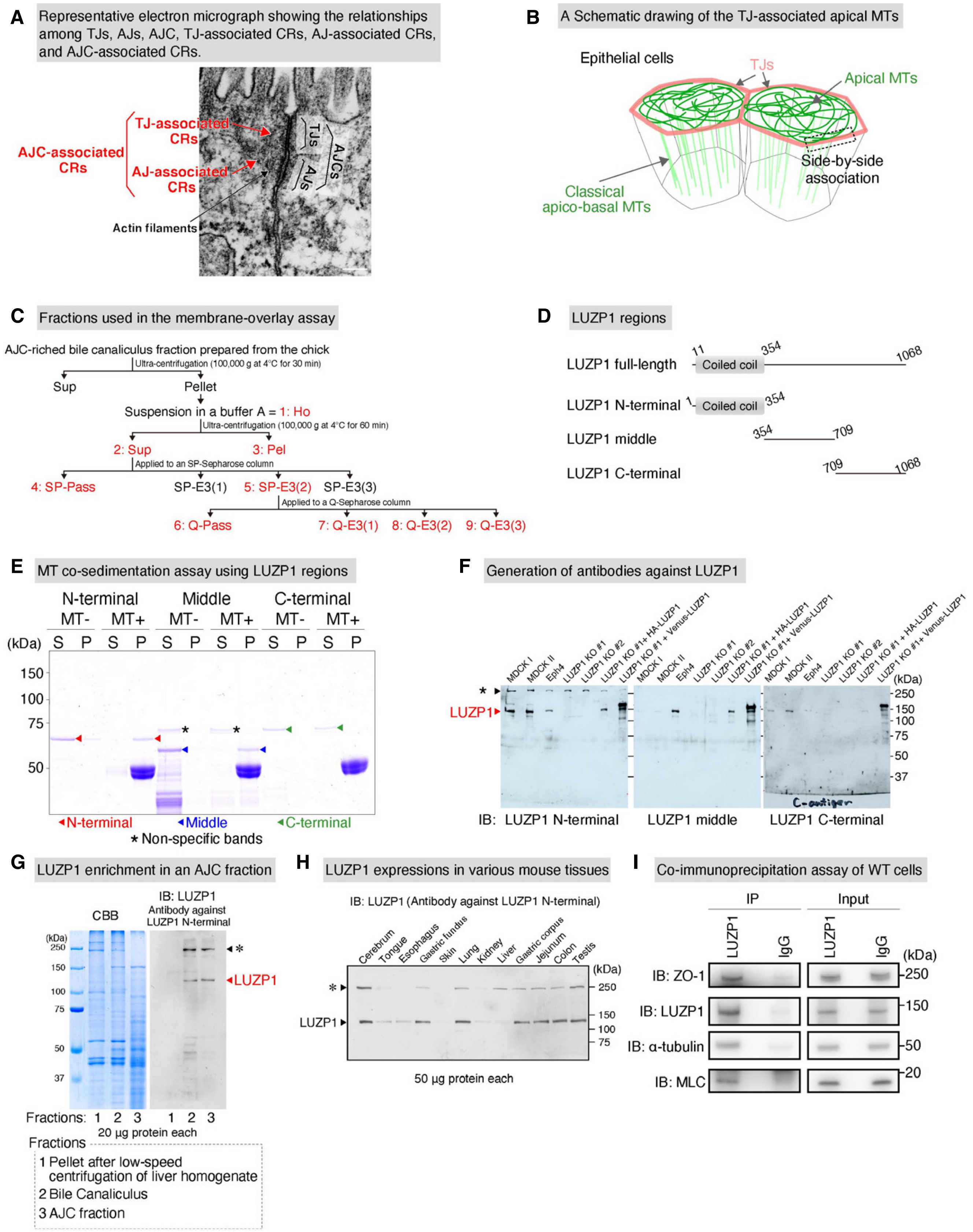
A Representative electron micrograph showing the relationships among TJs, AJs, AJC, TJ-associated CRs, AJ-associated CRs, and AJC-associated CRs.
AJC-associated CRs
TJ-associated CRs
AJ-associated CRs
Actin filaments
TJs
AJs
AJCs
B A Schematic drawing of the TJ-associated apical MTs
Epithelial cells
TJs
Apical MTs
Side-by-side association
Classical apico-basal MTs
C Fractions used in the membrane-overlay assay
AJC-riched bile canaliculus fraction prepared from the chick
Ultra-centrifugation (100,000 g at 4°C for 30 min)
Sup
Pellet
Suspension in a buffer A = 1: Ho
Ultra-centrifugation (100,000 g at 4°C for 60 min)
2: Sup
3: Pel
Applied to an SP-Sepharose column
4: SP-Pass
SP-E3(1)
5: SP-E3(2)
SP-E3(3)
Applied to a Q-Sepharose column
6: Q-Pass
7: Q-E3(1)
8: Q-E3(2)
9: Q-E3(3)
D LUZP1 regions
LUZP1 full-length
LUZP1 N-terminal
LUZP1 middle
LUZP1 C-terminal
Coiled coil
E MT co-sedimentation assay using LUZP1 regions
N-terminal
Middle
C-terminal
MT-
MT+
(kDa)
Non-specific bands
F Generation of antibodies against LUZP1
LUZP1
IB: LUZP1 N-terminal
LUZP1 middle
LUZP1 C-terminal
G LUZP1 enrichment in an AJC fraction
CBB
IB: LUZP1 Antibody against LUZP1 N-terminal
Fractions: 1 2 3
20 µg protein each
Fractions
1 Pellet after low-speed centrifugation of liver homogenate
2 Bile Canaliculus
3 AJC fraction
H LUZP1 expressions in various mouse tissues
IB: LUZP1 (Antibody against LUZP1 N-terminal)
Cerebrum
Tongue
Esophagus
Gastric fundus
Skin
Lung
Kidney
Liver
Gastric corpus
Jejunum
Colon
Testis
50 µg protein each
I Co-immunoprecipitation assay of WT cells
IP
Input
IB: ZO-1
IB: LUZP1
IB: α-tubulin
IB: MLC

Figure EV1.

Figure EV2. LUZP1 localizes at tight junction (TJ)-associated circumferential rings (CRs) in cultured epithelial cells and mouse tissues.

A Exogenous Venus-LUZP1 localization in Venus-LUZP1-expressing LUZP1 knockout (REV) Eph4 cells. Confocal micrographs of immunostained REV cells from the basal to apical planes showed that Venus-LUZP1 was also associated with cell–cell junctions at the level of TJs which are positive for ZO-1. Scale bar, 10 μm.

B Immunogold signals for TJ- and adherens junction (AJ)-related proteins in immunoelectron micrographs. Scale bars, 200 nm. MV, microvilli; DS, desmosome.

C Localization of LUZP1 in mouse embryonic neural tube. Confocal micrographs of immunostained E9.5 mouse embryos showed that, similar to cultured epithelial cells, LUZP1 was observed at cell–cell junctions. Scale bar, 50 μm.

D Localization of LUZP1 in mouse small intestine. Super-resolution micrographs of immunostained mouse small intestine showed that LUZP1 localized as two separate parallel lines along the single ZO-1-positive lines (arrows). This observation strongly suggests that, similar to cultured epithelial cells, LUZP1 also localizes at TJ-associated CRs in mouse tissues. Scale bar, 50 μm (low magnification) and 10 μm (high magnification).

E Representative confocal micrographs of co-cultures of wild-type (WT) and ZO-1/-2 double knockout (DKO) cells. ZO-1/-2 DKO cells were marked by asterisks (*). LUZP1 junctional localization was apparently disrupted in ZO-1/-2 DKO cells. Scale bar, 10 μm.

F Co-immunoprecipitation of LUZP1 and ZO-1, showing the binding of LUZP1 to ZO-1. IB, immunoblotting.

G Co-immunoprecipitation of LUZP1 and ZO-2, showing the binding of LUZP1 to ZO-2.

H Bar plots with dot density plots showing that LUZP1 knockout (KO) MTD-1A cells have significantly larger apical area than WT MTD-1A cells (42.9 ± 29.9 μm^2 [WT] vs. 102.3 ± 29.9 μm^2 [LUZP1 KO]) and LUZP1 KO CSG120/7 cells have significantly larger apical area than WT CSG120/7 cells (50.2 ± 24.8 μm^2 [WT] vs. 100.0 ± 36.6 μm^2 [LUZP1 KO]). $n = 3$. $^{**}P < 0.01$ (unpaired t-test). Bars and error bars represent the mean ± standard deviation (SD).

Source data are available online for this figure.

 © 2021 The Authors

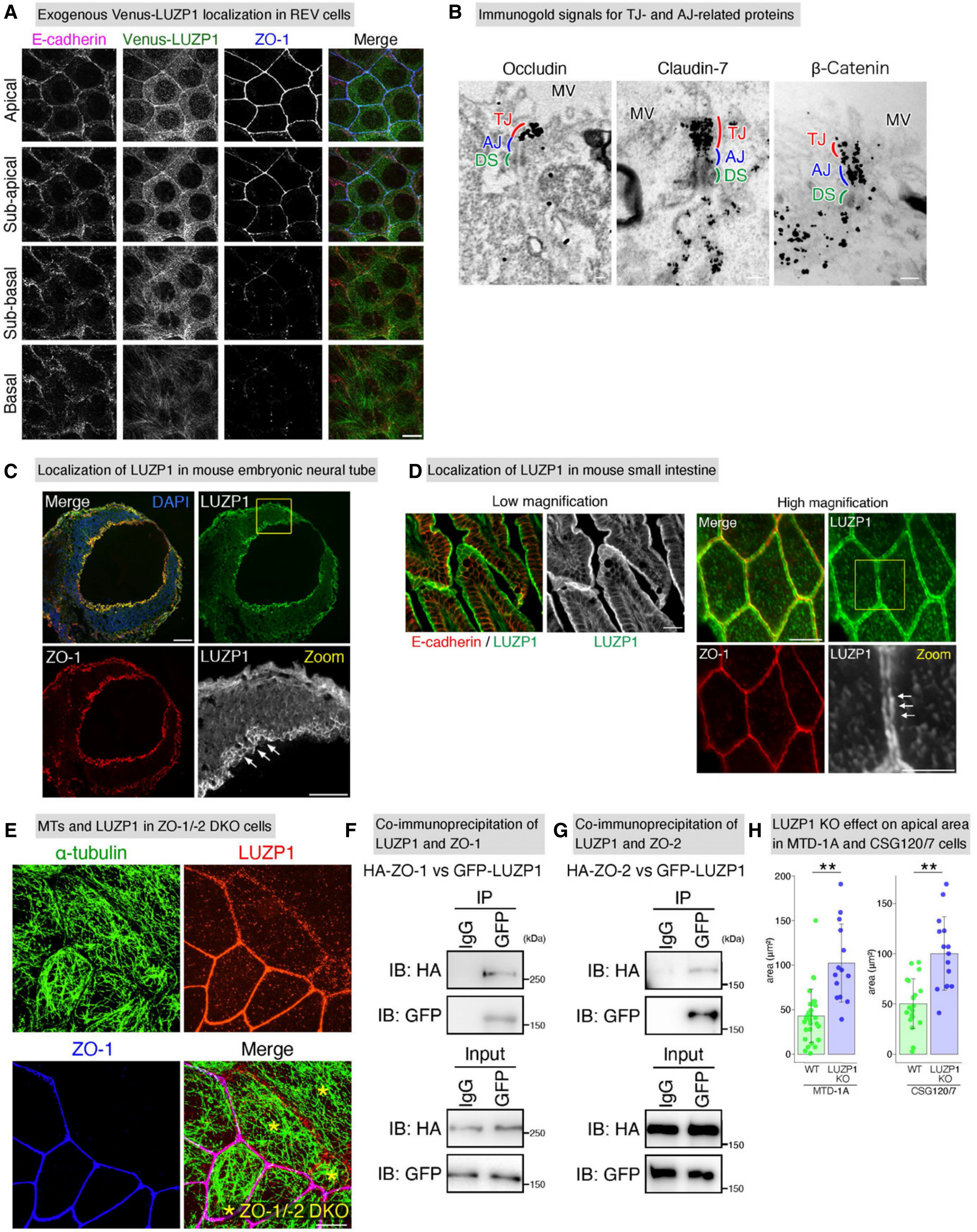
A
Exogenous Venus-LUZP1 localization in REV cells
E-cadherin
Venus-LUZP1
ZO-1
Merge
Apical
Sub-apical
Sub-basal
Basal
B
Immunogold signals for TJ- and AJ-related proteins
Occludin
Claudin-7
β-Catenin
MV
TJ
AJ
DS
C
Localization of LUZP1 in mouse embryonic neural tube
Merge
DAPI
LUZP1
ZO-1
LUZP1
Zoom
D
Localization of LUZP1 in mouse small intestine
Low magnification
High magnification
E-cadherin / LUZP1
LUZP1
Merge
LUZP1
ZO-1
LUZP1
Zoom
E
MTs and LUZP1 in ZO-1/-2 DKO cells
α-tubulin
LUZP1
ZO-1
Merge
* ZO-1/-2 DKO
F
Co-immunoprecipitation of LUZP1 and ZO-1
HA-ZO-1 vs GFP-LUZP1
IP
IgG
GFP
(kDa)
IB: HA
IB: GFP
Input
250
150
G
Co-immunoprecipitation of LUZP1 and ZO-2
HA-ZO-2 vs GFP-LUZP1
H
LUZP1 KO effect on apical area in MTD-1A and CSG120/7 cells
area (μm²)
WT
LUZP1 KO
MTD-1A
CSG120/7
**

Figure EV2.

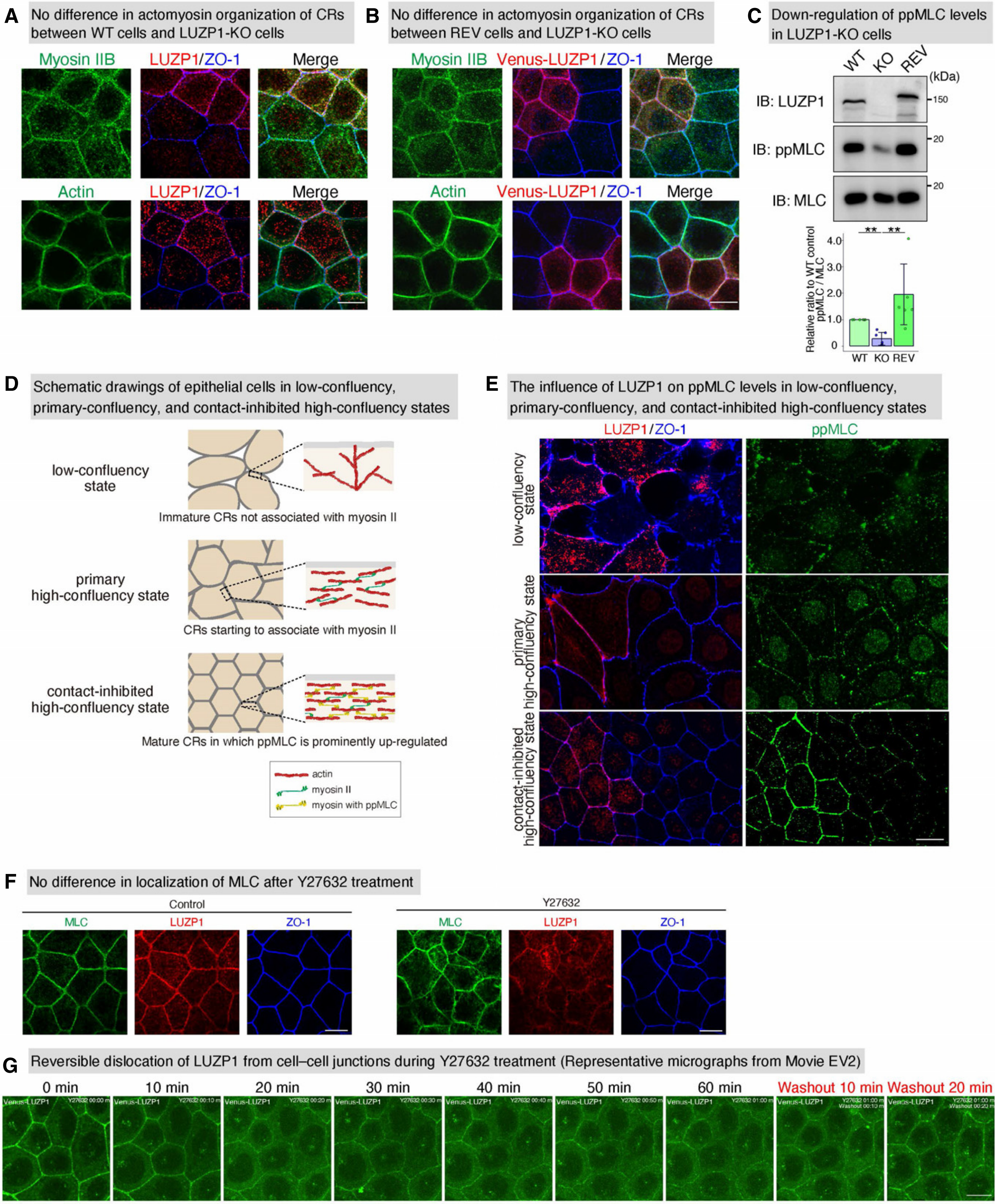

Figure EV3.

 © 2021 The Authors

Figure EV3. LUZP1 upregulates di-phosphorylated myosin light chain (ppMLC) levels within circumferential rings (CRs) especially in a contact-inhibited high-confluency state.

A No difference in actomyosin organization of CRs between wild-type (WT) and LUZP1 knockout (LUZP1 KO) Eph4 cells, shown by representative confocal micrographs of co-cultures of WT and LUZP1 KO Eph4 cells. Scale bar, 10 μm.
B No difference of actomyosin organization of CRs between Venus-LUZP1-expressing LUZP1 knockout (REV) and LUZP1 KO Eph4 cells shown by representative confocal micrographs of co-cultures of REV and LUZP1 KO Eph4 cells. Scale bar, 10 μm.
C Downregulation of ppMLC levels in LUZP1 KO Eph4 cells. Quantification of the ppMLC/MLC ratio relative to WT control revealed that ppMLC/MLC levels were significantly downregulated in LUZP1 KO Eph4 cells (1.00 [WT, control] vs. 0.30 ± 0.24 [KO] vs. 1.80 ± 1.18 [REV]). $n = 6$. $^{**}P < 0.01$ (Kruskal–Wallis test followed by Steel–Dwass test). Bars and error bars represent the mean ± standard deviation (SD). IB, immunoblotting.
D Schematic drawings of epithelial cells in low-confluency, primary-confluency, and contact-inhibited confluency states. In a low-confluency state when initial adhesion is made, epithelial cells have immature CRs not associated with myosin II. Even after a primary high-confluency state is established with CRs starting to have an association with myosin II, the cell number increases until a contact-inhibited high-confluency state, when ppMLC is prominently upregulated.
E The influence of LUZP1 on ppMLC levels in low-confluency, primary-confluency, and contact-inhibited high-confluency states. These representative confocal micrographs of co-cultures of WT and LUZP1 KO cells showed that the influence of LUZP1 to upregulate ppMLC levels was dependent on confluency states, with no influence being observed until a contact-inhibited high-confluency state. Scale bar, 10 μm.
F Representative confocal micrographs of WT Eph4 cells treated with 100 μM Y27632 for 30 min. The localization of MLC was not affected with transient Y27632 treatment. Scale bar, 10 μm.
G Representative micrographs from a live imaging of REV Eph4 cells treated with 100 μM Y27632 (Movie EV2). LUZP1 gradually dissociated from cell–cell junctions during Y27632 treatment, which was reversed by the washout of Y27632. Scale bar, 10 μm.

Source data are available online for this figure.

Figure EV4. LUZP1 inhibits protein phosphatase 1c β/δ (PP1c β/δ) in a microtubule (MT)-facilitated manner.

A No difference in Shroom3 localization between Venus-LUZP1-expressing LUZP1 knockout (REV) and LUZP1 knockout (LUZP1 KO) Eph4 cells, shown by representative confocal micrographs of co-cultures of WT and LUZP1 KO cells. Scale bar, 10 μm.
B Pull-down assays between Merlin and LUZP1, showing that Merlin did not bind LUZP1. IB, immunoblotting.
C No difference in MT organization between wild-type (WT) and LUZP1 KO Eph4 cells, shown by representative confocal micrographs of co-cultures of WT and LUZP1 KO Eph4 cells. Scale bar, 10 μm.
D No difference in α-tubulin intensities within circumferential rings (CRs) between WT and LUZP1 KO cells, shown by bar plots with dot density plots. (25.97 ± 4.69 arbitrary units [a.u.] [WT] vs. 27.03 ± 6.20 a.u. [KO]). $n = 3$. $P = 0.42$ (unpaired *t*-test). Bars and error bars represent the mean ± standard deviation (SD).
E Representative confocal micrographs of co-culture of Venus-LUZP1-expressing LUZP1 knockout (REV) and LUZP1 KO Eph4 cells treated with nocodazole in the apical and basal planes. Scale bar, 10 μm. Nocodazole treatment reversed the apical constriction of REV Eph4 cells. Scale bar, 10 μm.
F No difference in the degree of co-localization between LUZP1 and di-phosphorylated myosin light chain (ppMLC) after nocodazole treatment, shown by bar plots with dot density plots quantifying co-localization using Pearson's correlation coefficients (r) ($r = 0.48 \pm 0.12$ [control]; $r = 0.47 \pm 0.04$ [nocodazole]; $r = 0.49 \pm 0.12$ [washout]). $n = 3$. $P = 0.22$ (Kruskal–Wallis test). Bars and error bars represent the mean ± SD.
G *In vitro* Merlin phosphorylation assay using 1 μg MTs in addition to 100 ng GST-Merlin, 2 pg p21-activated kinase 1 (PAK1), 1 mM ATP, 1 μg GST- PP1c β/δ, and 0–5 μg GST-LUZP1, showing that MTs facilitated LUZP1-mediated inhibition of PP1c β/δ (1.00 [1st lane, control] vs. 1.73 ± 0.79 [2nd lane] vs. 3.03 ± 1.17 [3rd lane] vs. 6.06 ± 1.89 [4th lane] vs.2.69 ± 1.58 [5th-lane] vs. 4.45 ± 2.28 [6th lane] vs. 5.46 ± 2.68 [7th lane] vs. 8.32 ± 3.76 [8th lane]). $n = 5$. $^{*}P < 0.05$ (Kruskal–Wallis test followed by Steel test [compared with 1st lane]). Bars and error bars represent the mean ± SD.

Source data are available online for this figure.

© 2021 The Authors

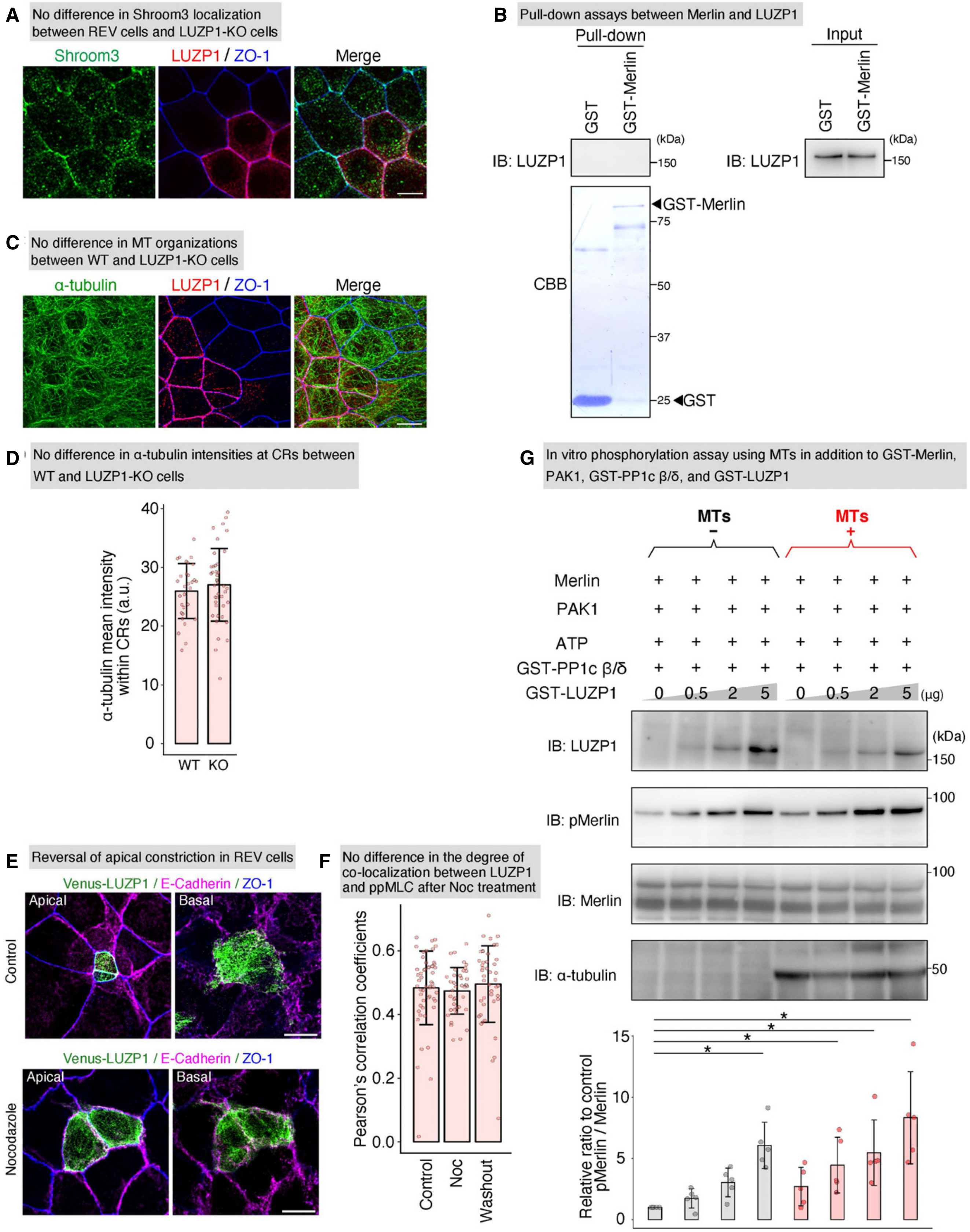

Figure EV4.

© 2021 The Authors
