# Supplementary material for: A microtubule‐LUZP1 association around tight junction promotes epithelial cell apical constriction
Source: EMBO J. 2020 Dec 21;40(2):e104712. doi: 10.15252/embj.2020104712 (PMC7809799; doi:10.15252/embj.2020104712)
Supplement: Supplementary file 3 — Movie EV1 [file EMBJ-40-e104712-s003.zip › Movie_EV1/Movie_EV1_legend.docx]

**Movie EV1. Localization of LUZP1 in cultured epithelial Eph4 cells.**

LUZP1 was preferentially associated with cell–cell junctions at the level of tight junctions which are positive for ZO-1.
