# Supplementary material for: A microtubule‐LUZP1 association around tight junction promotes epithelial cell apical constriction
Source: EMBO J. 2020 Dec 21;40(2):e104712. doi: 10.15252/embj.2020104712 (PMC7809799; doi:10.15252/embj.2020104712)

**Movie EV2. Live imaging of Venus-LUZP1-expressing LUZP1 knockout (REV) cells during 100 μM Y27632 treatment.**

Venus-LUZP1 gradually dissociated from tight junctions during Y27632 treatment and re-localized to cell–cell junctions after Y27632 washout.
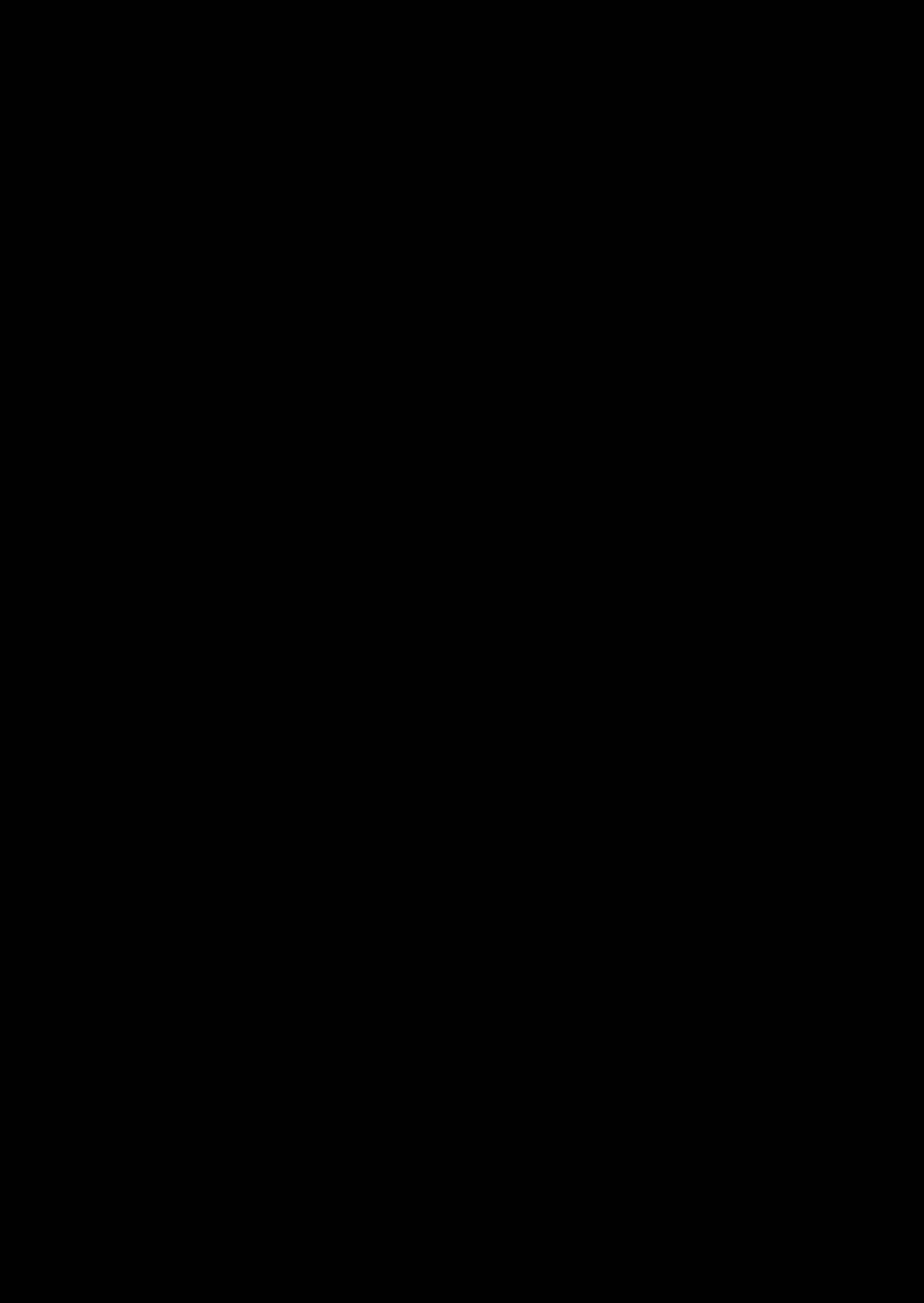

Supplement: Supplementary file 4 — Movie EV2 [file EMBJ-40-e104712-s004.zip › Movie_EV2/Movie_EV2_legend.docx]
