# Supplementary figures and images for: A microtubule‐LUZP1 association around tight junction promotes epithelial cell apical constriction
Source: EMBO J. 2020 Dec 21;40(2):e104712. doi: 10.15252/embj.2020104712 (PMC7809799; doi:10.15252/embj.2020104712)

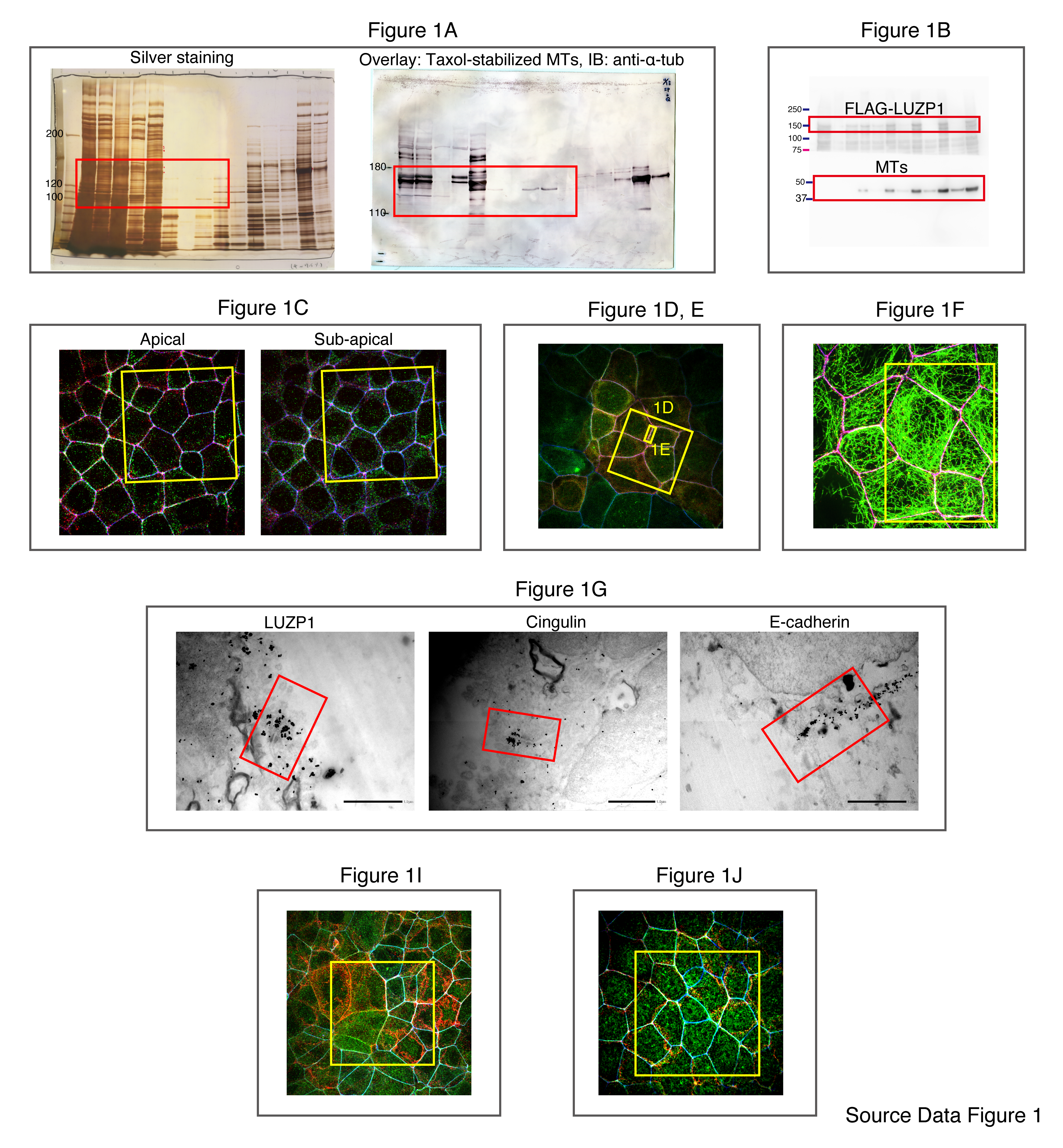

Supplement: Supplementary file 7 — Source Data for Figure 1 [file EMBJ-40-e104712-s005.tif]
